# Short-Term Cardiovascular Changes During the StressLess University Dog-Assisted Wellbeing Program: Evaluation of Integrated Cardiovascular Indices

**DOI:** 10.3390/healthcare14162494

**Published:** 2026-08-11

**Authors:** José Gijón, Meriem Khaled, María J. Lirola, Miguel C. Botella

**Affiliations:** 1 Department of Didactics and School Organization, University of Granada, 18071 Granada, Spain; meriemkg@ugr.es; 2 Department of Psychology, University of Almería, 04120 Almería, Spain; mariajesus.lirola@ual.es; 3 Department of Legal Medicine, Toxicology and Physical Anthropology, University of Granada, 18016 Granada, Spain; mbotella@ugr.es

**Keywords:** university students, dog-assisted wellbeing, cardiovascular response, systolic blood pressure, heart rate, physiological regulation, integrated cardiovascular indices, StressLess

## Abstract

**Background/Objectives**: Dog-assisted wellbeing activities are increasingly used in higher education, but their short-term cardiovascular correlates and the usefulness of integrated physiological indices remain insufficiently examined. This study evaluated cardiovascular changes associated with participation in StressLess, a multicomponent university wellbeing program involving supervised interaction with trained dogs, and explored several integrated cardiovascular indices. **Methods**: A total of 147 students participated voluntarily, generating 379 attendances, of which 375 complete session-level pre–post records were retained. Systolic blood pressure (SBP), diastolic blood pressure (DBP), and heart rate (HR) were measured before and after standardized rest periods and 30–40 min of supervised interaction. The primary analysis was restricted to each participant’s first attendance (N = 147) to ensure independence. Sensitivity analyses included all valid session records and participant-level averages. **Results**: At first attendance, mean SBP decreased from 117.56 to 112.88 mmHg, DBP from 74.05 to 70.95 mmHg, and HR from 82.11 to 75.09 beats/min (all *p* < 0.001; r = 0.41–0.59). The StressLess Cardiovascular Dynamics Index also decreased significantly (mean reduction = 0.408; *p* < 0.001; r = 0.66), and alternative formulations produced comparable results. Both sensitivity analyses reproduced the direction and statistical significance of the primary findings. **Conclusions**: Participation in the complete StressLess session was associated with short-term cardiovascular reductions. Because no control condition was included, the specific contribution of dog interaction cannot be separated from rest, social contact, expectancy, and temporary disengagement from academic activity. Integrated indices may provide useful exploratory summaries but require external validation before diagnostic or clinical use.

## 1. Introduction

### 1.1. Academic Stress and Dog-Assisted Interventions

Academic stress is one of the most prevalent challenges affecting university students worldwide and has been associated with a wide range of psychological, physiological, and academic consequences. Elevated stress levels have been linked to anxiety, emotional distress, reduced wellbeing, impaired academic performance, and an increased risk of academic disengagement and dropout [1,2,3,4].

University students frequently experience periods of heightened stress associated with examinations, assignment deadlines, academic workload, financial concerns, and the transition to independent living. Although moderate levels of stress may contribute to motivation and adaptation, persistent or excessive stress can negatively affect both mental and physical health, making the promotion of student wellbeing an increasingly important priority for higher education institutions [3,4].

In response to these concerns, universities have implemented a variety of wellbeing initiatives aimed at supporting students during periods of increased academic demand. Among these approaches, dog-assisted interventions have attracted growing attention due to their accessibility, positive acceptance among students, and potential capacity to reduce stress and anxiety in educational settings [5,6,7,8].

Previous studies have reported beneficial effects of interactions with therapy dogs on perceived stress, mood, anxiety, and physiological indicators of stress among university students [5,6]. Furthermore, systematic reviews and recent meta-analyses suggest that dog-assisted interventions may represent a valuable complementary strategy within broader university wellbeing programs [8]. Nevertheless, despite the growing evidence supporting these interventions, important methodological challenges remain regarding the objective assessment of their physiological effects.

### 1.2. Physiological Assessment of Stress

The assessment of stress in university students has traditionally relied on self-report measures focused on perceived stress, anxiety, emotional wellbeing, and related psychological constructs. Although these instruments provide valuable information about subjective experiences, they do not always correspond closely to physiological responses associated with stress activation [9].

From a psychophysiological perspective, stress involves coordinated changes across multiple biological systems, particularly the autonomic nervous system and the hypothalamic–pituitary–adrenal axis [10]. Among the physiological markers most frequently employed in stress research, blood pressure and heart rate have attracted considerable attention because they are non-invasive, inexpensive, and easily applicable in educational and community settings [11]. Physiological markers have also been increasingly incorporated into educational stress research [12].

Stress-related cardiovascular responses are commonly reflected in increases in systolic blood pressure, diastolic blood pressure, and heart rate, which provide useful indicators of autonomic activation and physiological arousal. However, these variables capture partially different aspects of cardiovascular functioning and may exhibit distinct response patterns across individuals and situations [9,13]. Consequently, the isolated interpretation of individual cardiovascular measures may not always provide a comprehensive representation of physiological stress responses.

These limitations have encouraged researchers to explore approaches capable of integrating multiple physiological signals in order to obtain more robust and informative indicators of stress and recovery processes. Such approaches are particularly relevant in applied contexts, where simple and practical methods are required to evaluate physiological changes associated with wellbeing interventions.

### 1.3. Integrated Cardiovascular Indicators

Recent developments in stress research have highlighted the growing importance of composite physiological indicators capable of integrating information from multiple biological measures. This perspective is consistent with multidimensional models of stress, such as allostatic load theory, which conceptualize physiological adaptation as the result of interactions among several biological systems rather than the activity of a single biomarker [14].

Recent reviews have further emphasized the potential value of combining physiological signals to improve the assessment of stress and recovery processes [15,16]. Integrated approaches may provide a more stable representation of physiological activation by reducing the influence of individual variability associated with isolated measures and by capturing broader patterns of cardiovascular response.

Several composite indicators have been proposed in the medical and physiological literature. Among the most widely used are mean arterial pressure (MAP), which combines systolic and diastolic blood pressure into a single estimate of circulatory load, and the rate-pressure product (RPP), an index reflecting the combined influence of blood pressure and heart rate on cardiovascular workload [17]. These approaches illustrate the broader methodological principle that integrated measures can provide additional information beyond that obtained from individual physiological variables considered separately.

Despite their potential advantages, integrated cardiovascular approaches remain relatively uncommon in educational and university wellbeing research. Most studies evaluating stress-reduction interventions continue to analyze blood pressure and heart rate independently, limiting the possibility of obtaining a unified representation of physiological responses.

In this context, the present study explores the usefulness of integrated cardiovascular indicators for monitoring physiological changes associated with dog-assisted university interventions. Rather than proposing a diagnostic tool, the aim is to examine whether different cardiovascular formulations can provide a practical and consistent framework for summarizing short-term cardiovascular response patterns in real educational settings.

### 1.4. Study Objectives

The primary objective of this study was to examine short-term changes in systolic blood pressure, diastolic blood pressure, and heart rate associated with participation in the complete StressLess university wellbeing session. Because the program combined supervised interaction with trained dogs, seated rest, informal social contact, and temporary disengagement from academic activity, the study evaluated the cardiovascular response to the session as a whole rather than attempting to isolate the specific effect of dog interaction.

The secondary objective was to explore whether integrated cardiovascular formulations could provide concise and consistent summaries of the joint pattern of change across the individual physiological measures. The StressLess Cardiovascular Dynamics Index (SCDI) was therefore compared with indices based on SBP and HR, mean arterial pressure and HR, and the standardized rate-pressure product. These analyses were intended to assess convergence and robustness across alternative methods of cardiovascular aggregation, not to establish the diagnostic validity or superiority of any particular index.

It was expected that participation would be associated with reductions in SBP, DBP, and HR and that the integrated indices would reflect a comparable overall direction of cardiovascular change. The primary analysis was based on participants’ first valid attendance to ensure independence of observations. Additional exploratory analyses examined the robustness of the findings across repeated attendances, the association between baseline cardiovascular levels and subsequent change, and possible differences between one-time and repeat attendees.

## 2. Materials and Methods

### 2.1. Study Design

A single-group pre–post design was used to examine short-term cardiovascular changes associated with participation in the StressLess program. Students were permitted to attend more than one session; however, the primary inferential analysis was restricted to each participant’s first valid attendance to ensure independence of observations. Repeated session records were retained for complementary sensitivity analyses conducted both at the session level and after aggregation within participants.

The study combined an applied objective—examining cardiovascular changes associated with participation in the complete StressLess session—with a secondary methodological objective exploring whether integrated cardiovascular indices could provide concise summaries of the joint response observed across systolic blood pressure, diastolic blood pressure, and heart rate.

StressLess was a multicomponent university wellbeing activity comprising supervised interaction with trained dogs, seated rest, informal social interaction with the handler and other students, and temporary disengagement from academic demands. Accordingly, the design evaluated the session as a whole and was not intended to isolate the specific contribution of dog interaction.

The study was conducted under routine university conditions and reflected the operational characteristics of a naturally occurring wellbeing program in higher education. It was not conceived as a clinical trial and did not include a control or comparison condition. The findings were therefore interpreted as short-term cardiovascular changes associated with participation rather than as evidence of a causal effect attributable to any individual component of the session.

The StressLess program was originally implemented within a university innovation and wellbeing initiative approved by the Human Research Ethics Committee of the University of Granada (Reference No. 1650/CEIH/2020). The present research evaluation, including the collection and analysis of cardiovascular data, was subsequently approved under Reference No. 3126/CEIH/2023. All participants provided written informed consent before participation, and all procedures complied with the principles of the Declaration of Helsinki.

### 2.2. Participants, Recruitment, and Attendance

A total of 147 university students voluntarily participated in the StressLess program and provided physiological measurements during the study period. Participants were recruited through institutional announcements and brief presentations conducted in university classes with the permission of the corresponding lecturers. The program was openly presented as a university wellbeing initiative involving interaction with trained dogs; no cover story or deception was used. Before participation, students received information about the purpose and procedures of the program, the voluntary nature of participation, the processing of their data, and their right to withdraw, and provided written informed consent.

The mean age of participants was 22.35 years (SD = 3.00), with ages ranging from 19 to 45 years. The sample comprised 125 women (85.0%) and 22 men (15.0%). Most participants were enrolled in the Social Education degree program (n = 127, 86.4%), whereas 20 participants (13.6%) were enrolled in the Master’s Degree in Secondary Education Teacher Training (MAES). The predominantly female composition of the sample reflected the demographic distribution of the academic programs from which participants were recruited.

Participation was voluntary and uncompensated. Inclusion criteria consisted of being enrolled as a university student, voluntarily registering for a StressLess session, and providing informed consent. Participants were not selected according to dog ownership, previous experience with dogs, or attitudes toward animals, and these characteristics were not systematically assessed.

Students could participate in more than one session, but no participant attended more than one session on the same day. Consequently, all repeated attendances occurred on different dates. Of the 147 participants, 29 attended one session, 5 attended two sessions, 112 attended three sessions, and 1 participant attended four sessions.

A total of 379 session attendances were initially recorded. Four session-level records were excluded during data cleaning: two because complete pre–post physiological measurements were unavailable and two because heart rate readings were judged physiologically implausible during data verification. The final dataset therefore comprised 375 complete session-level pre–post records, corresponding to 750 physiological measurement occasions. Each valid session-level record included systolic blood pressure (SBP), diastolic blood pressure (DBP), and heart rate (HR) measured before and after participation in a StressLess session.

### 2.3. StressLess Program and Intervention Procedure

The study was conducted within the StressLess program, a university wellbeing initiative implemented at the Faculty of Education Sciences of the University of Granada. The sessions were scheduled during periods of increased academic demand and were organized in relation to participants’ examination calendars. For students attending successive sessions, the final session was generally held on the day preceding an examination, whereas earlier sessions were scheduled approximately one or two weeks beforehand.

All sessions took place in the same room within the Faculty of Education Sciences Library. The room was reserved exclusively for the program and was visually and acoustically separated from the remaining library areas. The use of a constant physical setting was intended to reduce environmental variation between sessions while maintaining the ecological characteristics of a university wellbeing activity.

Sessions were offered at 10:30, 11:30, and 12:30 on Mondays, Wednesdays, and Fridays, depending on student availability and the schedule of the animal-assisted intervention provider. Each group comprised between three and eight students, with an average of approximately five participants. Two or three dogs were present depending on group size, maintaining an approximate ratio of two to three students per dog. A representative image of the session setting and supervised student–dog interaction is provided in Supplementary Figure S1.

The intervention dogs—Pecas, Garbanzo, and Rufino—belonged to Hachiko Educación Canina and had been trained for animal-assisted activities in educational and healthcare contexts and with people with intellectual or physical disabilities. The same dogs and the same professional handler participated throughout the program. This consistency was intended to limit between-session variability associated with the individual dogs or the person facilitating the intervention.

Each complete session lasted approximately 60 min and followed a standardized sequence. After arrival, participants remained seated for an initial 10-min period before the first physiological measurements were obtained. They then interacted with the dogs and the other students for approximately 30–40 min. The intended interaction period was 30 min, although it was occasionally extended slightly to allow the activity to end naturally and to avoid rushing participants immediately before the post-session assessment. Following the interaction, participants remained seated for a second 10-min period before the final physiological measurements were collected.

The dog-assisted component was free but supervised rather than based on a fixed sequence of therapeutic tasks. Participants sat or lay on floor mats beside the dogs, petted them, maintained calm physical contact, and engaged in informal conversation with the handler and the other students. Conversations commonly concerned the dogs, their characteristics, training, and activities. Participants were encouraged to maintain a calm atmosphere and to avoid discussing examinations or other academic demands during the intervention period.

The handler facilitated contact between the students and the dogs, monitored the animals’ behavior and welfare, and adapted the distribution of the dogs within the group when necessary. The intervention was not conceived as clinical therapy, but as a structured university wellbeing activity designed to provide a calm break from academic demands through supervised interaction with trained dogs and fellow students.

### 2.4. Physiological Measurement Procedure

Systolic blood pressure (SBP), diastolic blood pressure (DBP), and heart rate (HR) were assessed immediately before and after the dog-assisted interaction using four OMRON RS2 automatic wrist blood pressure monitors (OMRON Healthcare Co., Ltd., Kyoto, Japan). Before the beginning of data collection, the four devices were cross-checked against a conventional sphygmomanometer in an independent group of 30 adults who did not participate in the study. The observed discrepancies were small, generally approximately 1–2 mmHg for blood pressure measurements and 1–2 beats per minute for heart rate.

Measurements were self-administered under the direct supervision of one or two members of the research team. Before measurement, participants received instructions on the correct use of the device and on the standardized measurement position. Participants remained seated, with their back and feet supported and the wrist positioned approximately at heart level. They were instructed not to speak or move during the measurement. The same wrist and, whenever possible, the same device were used for the pre- and post-session assessments of each participant.

One reading was normally recorded at each assessment point. When a value appeared implausible or outside the expected range, the measurement was repeated in accordance with the manufacturer’s instructions. Another student recorded the displayed values on a session-specific data sheet under the supervision of the research team. Each sheet included the participant’s anonymized identification code, date, attendance number, and pre- and post-session SBP, DBP, and HR values.

The initial measurement was obtained after participants had remained seated for approximately 10 min. This rest period was included to minimize the influence of recent physical activity, including walking through the building or climbing the stairs leading to the library. During this period, participants completed a shortened version of the State-Trait Anxiety Inventory; these psychological data were not included in the present study. Following the 30–40-min dog-assisted interaction, participants again remained seated for approximately 10 min while completing the same short questionnaire. The post-session cardiovascular measurements were then obtained following the same procedure as the initial assessment.

### 2.5. Construction of Integrated Cardiovascular Indices

To explore whether composite measures could provide concise summaries of the overall cardiovascular response, several integrated indices were constructed from systolic blood pressure (SBP), diastolic blood pressure (DBP), and heart rate (HR) measurements obtained before and after each StressLess session.

Because the cardiovascular variables were expressed in different units and measurement ranges, standardized scores (z-scores) were calculated to facilitate their integration. For each variable, the pre- and post-session values were standardized jointly using the pooled mean and standard deviation calculated across all 750 valid pre- and post-session measurement sets in the complete dataset. These fixed reference parameters were retained in the primary and sensitivity analyses to ensure that the resulting index values remained directly comparable across analytical samples. Standardization was performed using the conventional formula:

z=\frac{x−μ}{σ}

where *x* represents the observed value, *μ* the pooled mean calculated from the complete dataset, and *σ* the corresponding pooled standard deviation.

Based on these standardized values, the StressLess Cardiovascular Dynamics Index (SCDI) was calculated separately for the pre- and post-session assessments as the arithmetic mean of the standardized SBP, DBP, and HR scores:

\mathrm{SCDI}=\frac{z_{\mathrm{SBP}} +z_{\mathrm{DBP}} +z_{\mathrm{HR}}  }{3}

The SCDI was conceived as an exploratory composite indicator intended to summarize the overall pattern of cardiovascular activation represented by three commonly used physiological measures. It was not intended as a diagnostic instrument or as a substitute for the interpretation of the individual cardiovascular variables.

To examine whether the findings depended on the particular formulation selected, three additional cardiovascular indices were calculated and compared with the SCDI. The first consisted of a combined SBP + HR Index, obtained by averaging the standardized systolic blood pressure and heart rate values:

\mathrm{SBP+HR\ Index}=\frac{z_{\mathrm{SBP}}+z_{\mathrm{HR}}}{2}

The second formulation combined mean arterial pressure (MAP) and heart rate. MAP was calculated using the conventional expression:

\mathrm{MAP}=\frac{\mathrm{SBP}+2(\mathrm{DBP})}{3}

The MAP values were subsequently standardized jointly across the pre- and post-session observations, and a MAP + HR Index was obtained by averaging the standardized MAP and HR values:

\mathrm{MAP+HR\ Index}=\frac{z_{\mathrm{MAP}}+z_{\mathrm{HR}}}{2}

Finally, the Rate-Pressure Product (RPP) was calculated as a conventional indicator of cardiovascular workload:

\mathrm{RPP}=\frac{\mathrm{SBP}\times\mathrm{HR}}{100}

For comparability with the other integrated formulations, the pre- and post-session RPP values were also standardized using the pooled mean and standard deviation calculated across all 750 valid RPP observations. The standardized pre–post RPP change score was then calculated using the same direction as the other indices, with positive values representing lower cardiovascular workload after the session.

All integrated indices were calculated separately for the pre- and post-session measurements. The same fixed standardization parameters derived from the complete dataset were applied to the primary first-attendance sample and to both sensitivity analyses. The comparison of the alternative formulations was intended to examine convergence and robustness across related methods of cardiovascular aggregation. It was not intended to establish diagnostic validity, clinical applicability, or the superiority of any individual index.

### 2.6. Statistical Analysis

Statistical analyses were conducted using IBM SPSS Statistics, Version 26.0 (IBM Corp., Armonk, NY, USA), and Python, Version 3.13.5. Data verification, sensitivity and association analyses, and graphical representations were performed in Python using pandas, Version 2.2.3; SciPy, Version 1.17.0; and Matplotlib, Version 3.10.8. Data were examined for completeness and physiological plausibility before analysis. No missing values were imputed. Session-level records lacking the complete set of pre- and post-session cardiovascular measurements or containing heart rate values considered physiologically implausible during data verification were excluded.

Descriptive statistics were calculated for all physiological variables and integrated cardiovascular indices. These included means, standard deviations, medians, interquartile ranges, and observed ranges, as appropriate. Cardiovascular change scores were calculated as the pre-session value minus the post-session value; consequently, positive values indicated a reduction following the intervention. Distributional assumptions were examined using the Shapiro–Wilk test and through visual inspection of the data. Because several variables departed from normality, non-parametric procedures were used for the principal pre–post comparisons.

To ensure independence of observations, the primary inferential analysis was restricted to each participant’s first valid attendance, yielding one complete pre–post pair for each of the 147 participants. Pre–post differences in systolic blood pressure (SBP), diastolic blood pressure (DBP), heart rate (HR), and the integrated cardiovascular indices were evaluated using the Wilcoxon signed-rank test for paired samples.

Effect sizes for the Wilcoxon signed-rank tests were calculated using the absolute value of the standardized test statistic:

r=\frac{|Z|}{\sqrt{N}}

where |Z| represents the absolute value of the standardized Wilcoxon test statistic and N represents the total number of paired observations included in the corresponding analysis. Effect sizes were interpreted according to conventional criteria, with values of approximately 0.10, 0.30, and 0.50 representing small, medium, and large effects, respectively [18].

Two sensitivity analyses were conducted to examine the robustness of the primary findings. First, the pre–post comparisons were repeated using all 375 valid session-level records. Because some participants contributed measurements from more than one attendance, this analysis was interpreted as a session-level robustness check rather than as independent confirmatory evidence. Second, all valid attendances were averaged within each participant to obtain one participant-level mean pre-session value and one mean post-session value for each variable. These participant-level averages were then compared using Wilcoxon signed-rank tests, thereby retaining information from repeated attendances while giving equal weight to each participant.

To explore whether students who attended only once differed from those who attended repeatedly, participants were classified as one-time attendees or repeat attendees. Comparisons were based on measurements obtained during the first attendance. Age, baseline cardiovascular values, cardiovascular change scores, and SCDI change scores were compared using Mann–Whitney U tests. Sex and academic program distributions were compared using Fisher’s exact tests because of the small number of observations in some categories. These analyses were considered exploratory and were interpreted cautiously.

Associations between baseline cardiovascular values and the magnitude of pre–post change were examined using Spearman rank-order correlations. In addition, exploratory mean–difference plots following the Bland–Altman convention were generated for SBP, DBP, and HR. For each variable, the pre–post difference was plotted against the mean of the pre- and post-session measurements, and the mean difference and 95% limits of agreement were calculated as the mean difference ± 1.96 standard deviations. These analyses were used to examine whether the magnitude of change varied across the observed measurement range and were not intended to assess interchangeability between measurement methods.

Convergence among the integrated cardiovascular formulations was examined by calculating Pearson correlation coefficients between their pre–post change scores in the primary analytical sample. Correlations above 0.70 were interpreted as strong and correlations above 0.90 as very strong.

All statistical tests were two-sided, and statistical significance was established at *p* < 0.05. Exact p values are reported whenever informative, whereas values below 0.001 are presented as *p* < 0.001.

## 3. Results

### 3.1. Participant Attendance, Data Quality, and Analytical Samples

The 147 participants generated a total of 379 recorded session attendances. As shown in Table 1, 29 students attended once, 5 attended twice, 112 attended three times, and 1 student attended four times. All repeated attendances took place on different dates, and no participant attended more than one session on the same day.

Four session-level records were excluded during data cleaning. Two records lacked the complete set of pre- and post-session cardiovascular measurements, and two contained heart rate values considered physiologically implausible during data verification. No missing values were imputed. Following these exclusions, 375 complete session-level pre–post records were retained, comprising 147 valid first-session records, 117 second-session records, 110 third-session records, and 1 fourth-session record. These records corresponded to 750 cardiovascular measurement occasions.

The primary inferential sample comprised the first valid attendance of each participant and therefore included 147 independent pre–post pairs. Two complementary sensitivity samples were also examined: the complete set of 375 valid session-level records and a participant-level dataset in which all valid attendances were averaged within each of the 147 participants.

Visual inspection and Shapiro–Wilk tests indicated departures from normality for several cardiovascular variables and change scores. Consequently, Wilcoxon signed-rank tests were used for the primary and sensitivity pre–post comparisons, as specified in Section 2.6.

### 3.2. Primary Analysis: Cardiovascular Changes at First Attendance

The primary analysis included the first valid attendance of each of the 147 participants, thereby ensuring that each student contributed only one independent pre–post pair. Significant reductions were observed in all three cardiovascular variables following participation in the StressLess session.

Mean systolic blood pressure (SBP) decreased from 117.56 mmHg before the session to 112.88 mmHg after participation, corresponding to a mean reduction of 4.67 mmHg. Diastolic blood pressure (DBP) decreased from 74.05 to 70.95 mmHg, representing a mean reduction of 3.11 mmHg. Heart rate (HR) decreased from 82.11 to 75.09 beats per minute, corresponding to a mean reduction of 7.02 beats per minute.

Wilcoxon signed-rank tests indicated statistically significant pre–post differences for SBP (Z = −6.32, *p* < 0.001), DBP (Z = −5.01, *p* < 0.001), and HR (Z = −7.21, *p* < 0.001). The effect size was large for SBP (r = 0.52) and HR (r = 0.59) and moderate for DBP (r = 0.41). The largest cardiovascular change was therefore observed for heart rate.

These results indicate that the cardiovascular reductions observed in the complete session-level sensitivity analysis were also present when the analysis was restricted to one independent observation per participant. The corresponding primary pre–post results are summarized in Table 2.

**Figure 1:**
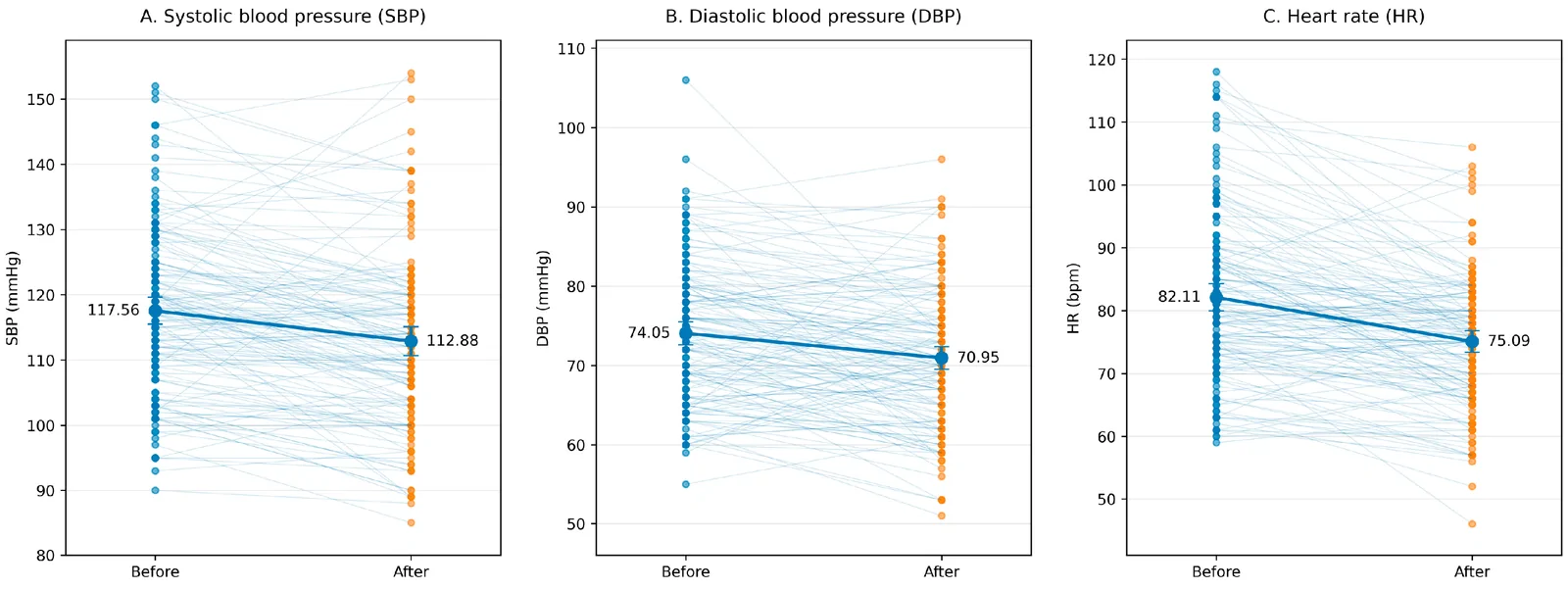
Individual cardiovascular measurements before and after the StressLess session at participants’ first attendance (N = 147). Each line connects the before- and after-session measurements of one participant. Large markers and error bars represent the mean and 95% confidence interval. Panels show (**A**) systolic blood pressure, (**B**) diastolic blood pressure, and (**C**) heart rate. SBP = systolic blood pressure; DBP = diastolic blood pressure; HR = heart rate. Blue markers represent before-session values, and orange markers represent after-session values.

Individual pre–post measurements at participants’ first attendance are displayed in Figure 1, showing both the overall direction of change and the variability in individual cardiovascular responses.

### 3.3. Sensitivity Analyses

Two complementary sensitivity analyses were conducted to determine whether the cardiovascular findings depended on restricting the primary analysis to participants’ first attendance.

First, the pre–post comparisons were repeated using all 375 valid session-level records. This analysis reproduced the pattern observed in the primary independent sample. Mean systolic blood pressure decreased from 116.08 to 110.77 mmHg, corresponding to a mean reduction of 5.31 mmHg (Z = −10.07, *p* < 0.001, r = 0.52). Mean diastolic blood pressure decreased from 74.06 to 70.44 mmHg, with a mean reduction of 3.62 mmHg (Z = −9.64, *p* < 0.001, r = 0.50). Mean heart rate decreased from 82.46 to 75.67 beats per minute, corresponding to a mean reduction of 6.78 beats per minute (Z = −11.83, *p* < 0.001, r = 0.61). Because these records included repeated attendances from some participants, the findings were interpreted as a session-level robustness check rather than as independent confirmatory evidence.

Second, all valid attendances were averaged within each participant, resulting in one participant-level mean before value and one participant-level mean after value for each cardiovascular variable. This approach retained information from repeated attendances while ensuring that each of the 147 participants contributed equally to the analysis. Mean systolic blood pressure decreased from 117.09 to 111.85 mmHg (mean reduction = 5.24 mmHg; Z = −7.69, *p* < 0.001, r = 0.63). Mean diastolic blood pressure decreased from 74.74 to 71.34 mmHg (mean reduction = 3.40 mmHg; Z = −7.26, *p* < 0.001, r = 0.60), and mean heart rate decreased from 83.31 to 75.60 beats per minute (mean reduction = 7.71 beats per minute; Z = −8.99, *p* < 0.001, r = 0.74).

Taken together, the two sensitivity analyses showed that the direction and statistical significance of the cardiovascular changes remained consistent when all valid session-level records were considered and when repeated attendances were aggregated at the participant level. The corresponding sensitivity analyses for the integrated cardiovascular indices are presented in Section 3.6. The results of these sensitivity analyses are summarized in Table 3.

### 3.4. Baseline Levels and Magnitude of Cardiovascular Change

The magnitude of cardiovascular change varied across participants. At the first attendance, individual SBP change scores ranged from a 35 mmHg increase to a 32 mmHg reduction, DBP change scores ranged from a 26 mmHg increase to a 32 mmHg reduction, and HR change scores ranged from an 18 beats-per-minute increase to a 39 beats-per-minute reduction. Positive change scores represent lower values after participation, whereas negative scores indicate that the cardiovascular value increased.

Higher baseline cardiovascular values were associated with larger subsequent reductions. Spearman rank-order correlations between baseline level and pre–post reduction were statistically significant for SBP (ρ = 0.334, *p* < 0.001), DBP (ρ = 0.352, *p* < 0.001), and HR (ρ = 0.580, *p* < 0.001). The association was strongest for heart rate, suggesting that participants with higher initial HR generally showed larger decreases during the session.

Because correlations between a baseline value and a change score may be influenced by mathematical coupling and regression to the mean, complementary mean–difference analyses were performed. The association between the average of the pre- and post-session measurements and the corresponding difference was negligible for SBP (ρ = −0.018, *p* = 0.829) and DBP (ρ = −0.029, *p* = 0.723). For HR, a modest positive association was observed (ρ = 0.265, *p* = 0.001), indicating that larger reductions in heart rate were somewhat more frequent among participants with higher average HR levels.

The mean–difference plots showed no clear proportional pattern across the observed SBP or DBP ranges. The mean reduction and 95% limits of agreement were 4.67 mmHg (−14.37 to 23.72) for SBP, 3.11 mmHg (−12.09 to 18.30) for DBP, and 7.02 beats per minute (−13.11 to 27.15) for HR. These limits describe the variability of individual pre–post changes and should not be interpreted as clinical thresholds.

The corresponding mean–difference plots for SBP, DBP, and HR are presented in Supplementary Figure S2. These results are summarized in Table 4.

### 3.5. Comparison Between One-Time and Repeat Attendees

Exploratory analyses compared participants who attended only one StressLess session (n = 29) with those who attended two or more sessions (n = 118). All comparisons were based on data obtained during participants’ first attendance, before their subsequent attendance pattern had developed.

One-time attendees were younger than repeat attendees (median age = 21 versus 22 years; U = 1044.5, *p* < 0.001). The groups did not differ significantly in sex distribution (Fisher’s exact *p* = 0.569). However, academic program distribution differed (Fisher’s exact *p* = 0.014): all one-time attendees were Social Education students, whereas the repeat-attendance group included 98 Social Education students and all 20 MAES students.

At their first attendance, one-time attendees presented higher baseline cardiovascular values than repeat attendees. Median baseline SBP was 122.0 mmHg among one-time attendees and 115.5 mmHg among repeat attendees (U = 2369.0, *p* = 0.001). Median baseline DBP values were 77.0 and 72.5 mmHg, respectively (U = 2366.0, *p* = 0.001), while median baseline HR values were 87.0 and 78.5 beats per minute, respectively (U = 2258.0, *p* = 0.008).

The magnitude of SBP reduction did not differ significantly between one-time and repeat attendees (median reductions = 8.0 and 4.0 mmHg, respectively; U = 1908.5, *p* = 0.337). No significant difference was observed for DBP reduction (median = 4.0 mmHg in both groups; U = 1557.0, *p* = 0.454) or for SCDI reduction (median = 0.600 and 0.336, respectively; U = 1979.0, *p* = 0.193). In contrast, the reduction in HR was greater among one-time attendees than among repeat attendees (median reductions = 12.0 and 5.0 beats per minute, respectively; U = 2285.0, *p* = 0.005).

Overall, repeat attendance was not associated with a uniformly larger or smaller cardiovascular response. These findings should be interpreted cautiously because attendance frequency was self-selected and the groups also differed in age and academic program composition. The exploratory comparisons are summarized in Table 5.

### 3.6. Integrated Cardiovascular Indices and Convergence

In the primary analysis based on participants’ first attendance (N = 147), all integrated cardiovascular indices showed statistically significant reductions after participation in the StressLess session (Table 6).

The StressLess Cardiovascular Dynamics Index (SCDI) decreased from a mean standardized value of 0.246 before the session to −0.162 after participation, corresponding to a mean reduction of 0.408 (Z = −7.97, *p* < 0.001). The associated effect size was large (r = 0.66).

The alternative formulations produced closely comparable results. The SBP + HR Index showed a mean reduction of 0.440 (Z = −7.74, *p* < 0.001, r = 0.64), the MAP + HR Index showed a mean reduction of 0.461 (Z = −8.02, *p* < 0.001, r = 0.66), and the standardized Rate-Pressure Product showed a mean reduction of 0.584 (Z = −7.89, *p* < 0.001, r = 0.65).

These findings indicate that all four composite formulations captured the overall pattern of cardiovascular change observed in the individual SBP, DBP, and HR measures. The integrated indices produced large and highly similar effect sizes, but the analyses were not designed to establish the diagnostic or predictive superiority of any particular formulation.

Sensitivity analyses reproduced the same pattern. When all 375 valid session-level records were analyzed, effect sizes ranged from r = 0.66 for the SBP + HR Index to r = 0.69 for the MAP + HR Index. When repeated attendances were averaged within each participant, effect sizes ranged from r = 0.76 to r = 0.80. All comparisons remained statistically significant at *p* < 0.001.

The pre–post change scores of the different integrated formulations were strongly correlated in the primary analytical sample. The SCDI correlated with the SBP + HR Index at r = 0.85, with the MAP + HR Index at r = 0.98, and with the standardized RPP at r = 0.84 (all *p* < 0.001). The particularly high correlation between the SCDI and the MAP + HR Index reflects the substantial overlap between formulations incorporating blood pressure and heart rate information.

Overall, the results demonstrate convergence and robustness across alternative methods of cardiovascular aggregation. However, in the absence of an external psychological, behavioral, or clinical criterion, the present findings should be interpreted as evidence that the indices describe a common cardiovascular response pattern rather than as validation of one index as superior to the others.

## 4. Discussion

### 4.1. Cardiovascular Changes Associated with the StressLess Session

The present study identified significant short-term reductions in systolic blood pressure, diastolic blood pressure, and heart rate following participation in the StressLess session. Importantly, these findings were observed in the primary analysis restricted to participants’ first attendance, in which each of the 147 students contributed only one independent pre–post pair. The same direction of change was reproduced in two sensitivity analyses: one using all 375 valid session-level records and another based on participant-level averages across repeated attendances. This consistency indicates that the main findings were not dependent on the inclusion of repeated observations.

At first attendance, mean systolic blood pressure decreased by 4.67 mmHg, diastolic blood pressure by 3.11 mmHg, and heart rate by 7.02 beats per minute. The effects were large for systolic blood pressure and heart rate and moderate for diastolic blood pressure. From an applied perspective, the relevance of these findings lies primarily in the consistent short-term modulation of several cardiovascular indicators within a routine university wellbeing program. The study was not designed to determine clinical changes in cardiovascular health, and the observed reductions should therefore be interpreted as indicators of temporary physiological regulation rather than as evidence of a therapeutic cardiovascular effect.

The findings are consistent with previous research reporting reductions in perceived stress, anxiety, and physiological arousal following dog-assisted interventions in higher education settings [5,6,7,8]. The present study extends this evidence by demonstrating that the cardiovascular pattern remained statistically significant when the analysis was limited to one independent observation per participant and when alternative approaches to repeated attendance were examined.

Heart rate showed the largest reduction among the individual cardiovascular variables. Participants with higher initial heart rates also tended to show larger decreases. This association remained modestly present in the complementary mean–difference analysis, whereas no comparable proportional pattern was observed for systolic or diastolic blood pressure. These findings suggest that heart rate may have been particularly responsive to the short-term conditions created during the session, although the study design does not allow the physiological mechanisms responsible for this response to be isolated.

The comparison between one-time and repeat attendees did not reveal a uniformly different cardiovascular response pattern. One-time attendees presented higher baseline cardiovascular values and showed a larger reduction in heart rate, but the groups did not differ significantly in systolic blood pressure, diastolic blood pressure, or SCDI reduction. Because attendance frequency was self-selected and the groups differed in age and academic program composition, these exploratory findings should not be interpreted as evidence that one group benefited more from the program.

The intervention should be understood as a multicomponent university wellbeing session rather than as an isolated exposure to dogs. Participants spent approximately 30–40 min interacting calmly with trained dogs, the professional handler, and fellow students, within a reserved space and following equivalent pre- and post-session rest periods. The observed cardiovascular changes may therefore reflect the combined contribution of human–dog interaction, temporary disengagement from academic demands, calm social interaction, physical rest, and the absence of screen-based activity. In the absence of comparison conditions involving rest only, peer interaction only, or dogs without group interaction, the specific contribution of each component cannot be determined.

Accordingly, the findings provide evidence that participation in the complete StressLess session was associated with short-term cardiovascular regulation. They do not establish that interaction with the dogs alone caused the observed reductions. Nevertheless, from an applied university perspective, the observed program-level response and practical feasibility of the complete program constitute relevant outcomes, even when the contribution of its individual components cannot yet be separated.

### 4.2. Interpretation and Value of the Integrated Cardiovascular Indices

The integrated cardiovascular analyses constituted a secondary methodological objective of the study. All four formulations—the SCDI, the SBP + HR Index, the MAP + HR Index, and the standardized RPP—showed significant pre–post reductions in the primary independent sample and produced closely comparable effect sizes. The same pattern was reproduced in the sensitivity analyses, indicating that the findings were not dependent on a single method of cardiovascular aggregation.

The SCDI was developed as a simple exploratory summary of standardized SBP, DBP, and HR values. Its effect size was slightly larger than those observed for the individual cardiovascular measures, although this difference should not be interpreted as proof that the index is intrinsically more sensitive. A larger effect may reflect the aggregation of several variables that changed in the same direction, rather than superior measurement performance. The principal physiological findings therefore remain the reductions observed in SBP, DBP, and HR, while the SCDI provides a complementary summary of their joint pattern.

The strong correlations between the SCDI and the alternative formulations indicate substantial convergence. This was particularly evident for the MAP + HR Index, which incorporates information closely related to the three components of the SCDI. Such correlations are therefore partly expected because the indices share underlying cardiovascular variables. Nevertheless, the consistency across formulations suggests that the overall conclusion of reduced cardiovascular activation was not an artifact of one specific mathematical expression.

Composite physiological measures may be useful in applied settings when researchers or practitioners require a concise representation of changes distributed across several indicators. This approach is consistent with multidimensional perspectives in which stress-related physiological responses are understood as coordinated patterns rather than as changes in a single biomarker [14,15,16]. However, aggregation also reduces the visibility of potentially different responses in the individual variables. Integrated indices should therefore complement, rather than replace, the separate interpretation of SBP, DBP, and HR.

The present study does not establish the diagnostic, predictive, or clinical validity of the SCDI or demonstrate that it is superior to the alternative indices. No external criterion—such as perceived stress, anxiety, behavioral response, or an independently validated physiological marker—was available against which the formulations could be compared. Future research should examine whether these indices are associated with psychological outcomes, distinguish different levels of stress, predict meaningful changes over time, and remain stable across populations and intervention settings. Until such evidence is available, the SCDI should be regarded as an exploratory descriptive tool for summarizing cardiovascular response patterns.

### 4.3. Practical Implications for University Wellbeing Programs

Universities require wellbeing initiatives that can be incorporated into everyday academic environments and offered during periods of increased demand. Within this context, the StressLess program represents a low-intensity and non-clinical activity intended to complement broader student-support strategies. The cardiovascular changes observed in this study suggest that participation in the complete session may provide a temporary period of physiological regulation. Such activities should not, however, be regarded as substitutes for psychological counseling, medical care, or other professional services required by students experiencing persistent or clinically significant difficulties.

The study also provides information relevant to the practical organization of similar programs. StressLess was implemented in a reserved and relatively quiet university library room, using small groups of three to eight students, two or three trained dogs, and the same professional handler throughout the intervention period. Sessions were scheduled around examinations and academic assessments and followed a stable sequence comprising pre-session rest, cardiovascular measurement, supervised interaction, post-session rest, and repeated measurement. This structured but relatively simple format may facilitate replication in universities with suitable spaces and access to qualified animal-assisted intervention professionals.

Implementation nevertheless requires more than merely allowing dogs into a university setting. Appropriate selection and preparation of the dogs, continuous supervision by a trained handler, monitoring of animal welfare, hygiene and risk-management procedures, informed consent, and respect for students who may have allergies, fear of dogs, cultural reservations, or no interest in participating are essential. Group size and the student-to-dog ratio must also be limited to prevent excessive stimulation or fatigue for the animals. Consequently, institutional feasibility should be evaluated in relation to available professional, spatial, ethical, and financial resources.

The multicomponent character of the session may itself be relevant to university practice. The program provided students with a scheduled interruption from academic activity, a calm environment, informal peer contact, interaction with the handler, and supervised contact with trained dogs. Universities may therefore view this type of activity not as an isolated animal exposure but as one possible form of structured restorative space embedded within a wider wellbeing strategy. Controlled comparative research is still needed to establish which components contribute most strongly to the observed response.

The collection of simple cardiovascular measures also proved feasible under routine educational conditions and may offer institutions an objective complement to satisfaction surveys and self-report questionnaires when evaluating short-term wellbeing activities. Nevertheless, these measures should be used for program evaluation rather than for individual screening, diagnosis, or clinical decision-making. Physiological data collection also requires standardized procedures, trained supervision, appropriate data protection, and careful communication so that students do not interpret normal short-term variability as evidence of a health problem.

Institutional decisions regarding adoption should therefore consider feasibility, student accessibility, animal welfare, professional oversight, and the quality of the evaluation design rather than relying solely on immediate pre–post changes. The present findings support further assessment of carefully structured dog-assisted sessions as one complementary component of university wellbeing provision, particularly during periods of elevated academic demand.

### 4.4. Future Research: Controlled, Longitudinal, and Multimodal Assessment

Future research should first address the causal limitations of the present pre–post design. Randomized or carefully matched controlled studies are needed to compare the complete StressLess session with alternative conditions such as quiet seated rest, informal peer interaction without dogs, contact with the handler without the animals, or another structured wellbeing activity. Such comparisons would help distinguish the contribution of dog interaction from the effects of physical rest, social contact, expectancy, temporary disengagement from academic demands, and participation in a calm environment.

Future studies should also incorporate validated psychological measures alongside cardiovascular outcomes. State anxiety, perceived stress, affect, and subjective relaxation could be assessed immediately before and after each session, while broader measures of academic stress and wellbeing could be collected across the examination period. Combining psychological and physiological information would allow researchers to determine whether cardiovascular reductions correspond to participants’ perceived emotional changes and would provide an external criterion for evaluating the SCDI and the alternative integrated indices.

The longitudinal effects of repeated participation also require investigation. The present sensitivity analyses showed that the overall pattern remained consistent when repeated attendances were considered, but the study was not designed to determine whether successive sessions produce cumulative, diminishing, or stable responses. Prospective designs with predetermined attendance schedules and multilevel statistical models could examine within-participant trajectories, dose–response patterns, and the persistence of any changes beyond the immediate post-session period.

A more detailed assessment of participant characteristics would also strengthen future research. Dog ownership, previous experience with animals, affinity toward dogs, expectations regarding the program, baseline stress, sleep, caffeine consumption, recent physical activity, medication use, and proximity to examinations may influence both participation and physiological response. Recording these variables would help evaluate self-selection and identify possible moderators without assuming that all students respond similarly to dog-assisted activities. Recruitment should also extend to more gender-balanced samples and to students from a wider range of academic disciplines and sociocultural contexts.

Recent research on human–pet touch further suggests that the frequency, duration, initiation, and type of contact may vary with wellbeing and mental-health status [19]. Future studies should therefore document these interaction characteristics and combine quantitative outcomes with qualitative accounts of perceived mechanisms, including restorative distraction, social connection, psychological safety, and perceptions of institutional care.

The integrated cardiovascular indices require further methodological evaluation before they can be considered validated measures. Future studies should assess their test–retest reliability, stability across settings, responsiveness relative to individual cardiovascular variables, and associations with validated psychological and physiological criteria. Comparisons with heart rate variability, electrodermal activity, respiratory measures, or endocrine biomarkers may clarify whether the indices capture a broader stress-regulation process or primarily summarize short-term cardiovascular change. The relative weighting of their components should also be examined rather than assuming that SBP, DBP, and HR contribute equally in every population or context.

Recent research has explored real-time stress-prediction models based on physiological data obtained from wearable devices [20]. Wearable sensors and multimodal analytical systems may eventually support more continuous and ecologically sensitive evaluation of student wellbeing interventions. Artificial intelligence and machine-learning methods could be useful when sufficiently large, diverse, and prospectively collected datasets become available. However, these approaches should follow—not replace—controlled research, external validation, and transparent statistical analysis. Their use would also require strict attention to informed consent, data minimization, privacy, algorithmic bias, interpretability, and the risk that wellbeing monitoring could be perceived as surveillance. Such systems should support research and program evaluation rather than automated diagnosis or individual decision-making.

Finally, future investigations should assess implementation outcomes alongside participant responses. These include attendance, acceptability, accessibility, cost, professional requirements, institutional feasibility, and the welfare and workload of the intervention dogs. A comprehensive evaluation of dog-assisted university programs must determine not only whether short-term changes occur, but also whether the activity can be delivered ethically, sustainably, and equitably within routine higher-education settings.

## 5. Limitations

Several limitations should be considered when interpreting the findings of this study. First, the study used a single-group pre–post design without a control or comparison condition. Consequently, the observed cardiovascular reductions cannot be attributed specifically to interaction with the dogs. The complete StressLess session also included seated rest, temporary disengagement from academic activities, informal peer interaction, contact with the professional handler, and participation in a calm environment. Expectancy effects, habituation to the measurement procedure, regression to the mean, and normal short-term physiological variability cannot be excluded. The findings therefore demonstrate changes associated with participation in the complete program but do not establish that the dogs alone caused those changes.

Second, participation was voluntary, and recruitment materials openly described StressLess as a dog-assisted wellbeing program. Students who chose to participate may therefore have had more positive attitudes toward dogs, stronger expectations of benefit, or greater interest in this type of activity than non-participants. Dog ownership, previous experience with animals, affinity toward dogs, fear of dogs, and expectations regarding the intervention were not measured. This potential self-selection limits the extent to which the findings can be generalized to students with neutral or negative attitudes toward dogs.

Third, the sample was recruited from a single university and was predominantly female, with women representing approximately 85% of participants. Most students were enrolled in Social Education, and the remainder were studying for the Master’s Degree in Secondary Education Teacher Training. Although this composition reflects the demographic characteristics of the participating academic programs, it limits generalization to more gender-balanced populations, other academic disciplines, universities, cultural settings, and age groups. The small number of men also prevented meaningful sex-stratified analyses.

Fourth, several factors capable of influencing cardiovascular measurements were not systematically recorded or controlled. These included baseline psychological stress, sleep quality, caffeine or nicotine consumption, medication use, recent food intake, physical activity before arrival, time since climbing the stairs to the library, menstrual-cycle phase, time of day, and proximity to specific examinations. Sessions were scheduled during periods of academic demand, but individual exposure to imminent assessments was not identical. These uncontrolled variables may have contributed to both baseline differences and variability in the pre–post response.

Fifth, the present analysis focused exclusively on SBP, DBP, and HR. A brief state-anxiety measure was administered as part of the operational session procedure, but psychological outcomes were not included in this study. It was therefore not possible to determine whether the cardiovascular reductions corresponded to changes in perceived stress, anxiety, affect, or subjective relaxation. The absence of an external psychological or physiological criterion also prevents formal validation of the integrated cardiovascular indices.

Sixth, cardiovascular measurements were obtained using automatic wrist devices under supervised field conditions. This approach increased feasibility and ecological applicability, and the devices were cross-checked against a conventional sphygmomanometer before the study. Nevertheless, wrist measurements are sensitive to posture and wrist position, and the cross-check did not constitute formal device validation or calibration. Measurements were generally based on a single reading before and after the session, with repetition only when a value appeared implausible or outside the expected operating range. Some residual measurement error and short-term within-person variability are therefore likely.

Seventh, some students attended more than one session. Restricting the primary analysis to the first valid attendance provided 147 independent pre–post pairs and addressed the principal concern regarding clustered observations. The participant-level averaged sensitivity analysis also gave equal weight to each student. However, the supplementary analysis of all 375 session-level records included repeated observations and should be interpreted only as a robustness check. Because attendance frequency was self-selected rather than experimentally assigned, the study cannot determine cumulative effects, dose–response relationships, or whether students who returned differed systematically in unmeasured characteristics.

Eighth, the physiological assessment was limited to measurements taken immediately before and after the session. No follow-up measurements were collected, and the duration of the observed cardiovascular changes is therefore unknown. The results indicate short-term regulation during the intervention period but provide no evidence of sustained reductions in stress, improvements in cardiovascular health, or longer-term academic or psychological benefits.

Finally, the SCDI and the alternative integrated formulations were exploratory descriptive indices derived from variables contained in the same dataset. Their strong correlations are partly expected because the formulations share underlying cardiovascular components. The study did not assess test–retest reliability, predictive validity, diagnostic accuracy, responsiveness relative to an independent criterion, or optimal component weighting. These indices should therefore not be interpreted as validated measures of stress or as tools for clinical screening and decision-making.

Despite these limitations, the consistency of the findings across the independent primary analysis and the complementary sensitivity analyses provides a useful basis for more rigorous controlled studies of the complete StressLess program and for further evaluation of integrated cardiovascular approaches in applied university settings.

## 6. Conclusions

Participation in the StressLess session was associated with significant short-term reductions in systolic blood pressure, diastolic blood pressure, and heart rate among university students during periods of academic demand. These changes were observed in the primary analysis based on one independent pre–post pair for each of the 147 participants and were reproduced in complementary sensitivity analyses using all valid session records and participant-level averages.

The findings should be attributed to the complete multicomponent session, which combined supervised interaction with trained dogs, seated rest, informal social contact, temporary disengagement from academic activity, and participation in a calm university environment. Because the study did not include a control condition, it cannot determine the specific contribution of the dogs or establish a causal effect of dog interaction alone. The observed reductions should therefore be interpreted as immediate cardiovascular changes associated with participation in the program rather than as evidence of clinical or lasting cardiovascular benefit.

The integrated cardiovascular indices produced consistent results across alternative formulations and provided concise summaries of the joint cardiovascular response. However, the individual SBP, DBP, and HR measures remain the principal physiological outcomes. The SCDI should be regarded as an exploratory descriptive index until its reliability, external validity, predictive value, and responsiveness have been examined in controlled studies incorporating psychological and additional physiological measures.

Overall, the results support further evaluation of carefully structured dog-assisted sessions as a complementary component of university wellbeing provision. Future research should use controlled and longitudinal designs, assess potential self-selection and participant expectations, include more diverse samples, and determine whether the observed short-term responses persist beyond the intervention period.

## Figures and Tables

**Table 1:** Participant characteristics and attendance pattern (N = 147).

Characteristic	Value
Age, mean (SD)	22.35 (3.00)
Age range	19–45
Women, n (%)	125 (85.0)
Men, n (%)	22 (15.0)
MAES students, n (%)	20 (13.6)
Social Education	127 (86.4%)
Attended one session, n (%)	29 (19.7)
Attended two sessions, n (%)	5 (3.4)
Attended three sessions, n (%)	112 (76.2)
Attended four sessions, n (%)	1 (0.7)
Total session attendances recorded	379
Session-level records excluded	4
Valid session-level pre–post records	375

MAES = Master’s Degree in Secondary Education Teacher Training. Each valid session-level record comprised one complete set of cardiovascular measurements obtained before and after the intervention. The attendance pattern refers to all 379 recorded attendances before the exclusion of four session-level records during data cleaning. The final analytical dataset included 375 complete session-level pre–post records.

**Table 2:** Primary pre–post analysis of cardiovascular variables at participants’ first attendance (N = 147).

Variable	Before, Mean	After, Mean	Mean Reduction	Z	*p*	r
SBP	117.56	112.88	4.67	−6.32	<0.001	0.52
(mmHg)						
DBP	74.05	70.95	3.11	−5.01	<0.001	0.41
(mmHg)						
HR	82.11	75.09	7.02	−7.21	<0.001	0.59
(bpm)						

Wilcoxon signed-rank tests. Positive mean reductions represent lower values after participation. SBP = systolic blood pressure; DBP = diastolic blood pressure; HR = heart rate; bpm = beats per minute.

**Table 3:** Sensitivity analyses of pre–post cardiovascular changes.

Analytical Sample	Variable	Before, Mean	After, Mean	Mean Reduction	Z	*p*	r
All valid session-level records (n = 375)	SBP	116.08	110.77	5.31	−10.07	<0.001	0.52
	(mmHg)						
	DBP	74.06	70.44	3.62	−9.64	<0.001	0.50
	(mmHg)						
	HR	82.46	75.67	6.78	−11.83	<0.001	0.61
	(bpm)						
Participant-level averages (N = 147)	SBP	117.09	111.85	5.24	−7.69	<0.001	0.63
	(mmHg)						
	DBP	74.74	71.34	3.40	−7.26	<0.001	0.60
	(mmHg)						
	HR	83.31	75.60	7.71	−8.99	<0.001	0.74
	(bpm)						

Wilcoxon signed-rank tests. Positive mean reductions represent lower values after participation. The analysis of all session-level records includes repeated attendances and is therefore interpreted as a robustness check. For the participant-level analysis, all valid attendances were averaged within each participant before conducting the paired comparison. SBP = systolic blood pressure; DBP = diastolic blood pressure; HR = heart rate; bpm = beats per minute.

**Table 4:** Magnitude of cardiovascular change and associations with baseline measurement level at first attendance (N = 147).

Variable	Mean Reduction	Observed Range of Reduction	Baseline–Reduction ρ	*p*	Mean Level–Difference ρ	*p*	95% Limits of Agreement
SBP	4.67	−35 to 32	0.334	<0.001	−0.018	0.829	−14.37 to 23.72
(mmHg)							
DBP	3.11	−26 to 32	0.352	<0.001	−0.029	0.723	−12.09 to 18.30
(mmHg)							
HR	7.02	−18 to 39	0.580	<0.001	0.265	0.001	−13.11 to 27.15
(bpm)							

Positive values represent reductions from before to after the session; negative values represent increases. Baseline–reduction associations and mean level–difference associations were calculated using Spearman rank-order correlations. Limits of agreement were calculated as the mean pre–post difference ± 1.96 standard deviations. SBP = systolic blood pressure; DBP = diastolic blood pressure; HR = heart rate; bpm = beats per minute.

**Table 5:** Exploratory comparison between one-time and repeat attendees at their first StressLess session.

Characteristic or Outcome	One-Time Attendees (n = 29)	Repeat Attendees (n = 118)	Test Statistic	*p*
Age, years, median (mean)	21.0 (21.03)	22.0 (22.68)	U = 1044.5	<0.001
Women, n (%)	26 (89.7)	99 (83.9)	Fisher’s exact test	0.569
Social Education, n (%)	29 (100.0)	98 (83.1)	Fisher’s exact test	0.014
Baseline SBP, mmHg, median (mean)	122.0 (124.21)	115.5 (115.92)	U = 2369.0	0.001
Baseline DBP, mmHg, median (mean)	77.0 (79.14)	72.5 (72.81)	U = 2366.0	0.001
Baseline HR, bpm, median (mean)	87.0 (88.00)	78.5 (80.66)	U = 2258.0	0.008
SBP reduction, mmHg, median (mean)	8.0 (5.55)	4.0 (4.46)	U = 1908.5	0.337
DBP reduction, mmHg, median (mean)	4.0 (2.34)	4.0 (3.30)	U = 1557.0	0.454
HR reduction, bpm, median (mean)	12.0 (12.97)	5.0 (5.56)	U = 2285.0	0.005
SCDI reduction, median (mean)	0.600 (0.552)	0.336 (0.373)	U = 1979.0	0.193

Repeat attendees participated in two or more StressLess sessions. All physiological comparisons refer exclusively to participants’ first attendance. Continuous variables were compared using Mann–Whitney U tests, and categorical distributions were compared using Fisher’s exact tests. Positive change values represent reductions from before to after the session. SBP = systolic blood pressure; DBP = diastolic blood pressure; HR = heart rate; bpm = beats per minute; SCDI = StressLess Cardiovascular Dynamics Index.

**Table 6:** Primary pre–post analysis of integrated cardiovascular indices at participants’ first attendance (N = 147).

Index	Before, Mean	After, Mean	Mean Reduction	Z	*p*	r
SCDI (SBP + DBP + HR)	0.246	−0.162	0.408	−7.97	<0.001	0.66
SBP + HR Index	0.269	−0.171	0.440	−7.74	<0.001	0.64
MAP + HR Index	0.254	−0.207	0.461	−8.02	<0.001	0.66
Standardized RPP	0.325	−0.258	0.584	−7.89	<0.001	0.65

Wilcoxon signed-rank tests. Positive mean reductions represent lower values after participation. All indices are expressed as standardized scores. SCDI = StressLess Cardiovascular Dynamics Index; SBP = systolic blood pressure; DBP = diastolic blood pressure; HR = heart rate; MAP = mean arterial pressure; RPP = rate-pressure product.

## Data Availability

The datasets generated and analyzed during the current study are not publicly available due to ethical and privacy considerations associated with the physiological data collected from participants. Data may be made available by the corresponding author upon reasonable request and subject to approval by the research team and compliance with applicable ethical and data protection requirements.

## Supplementary Materials

The following supporting information can be downloaded at: https://www.mdpi.com/article/10.3390/healthcare14162494/s1, Figure S1, Representative StressLess sessions conducted in the university library setting; Figure S2, Mean-difference plots for systolic blood pressure, diastolic blood pressure, and heart rate at participants’ first attendance.

## Abbreviations

The following abbreviations are used in this manuscript:

CI	Confidence Interval
DBP	Diastolic Blood Pressure
HR	Heart Rate
MAES	Master’s Degree in Secondary Education Teacher Training
MAP	Mean Arterial Pressure
RPP	Rate-Pressure Product
SBP	Systolic Blood Pressure
SCDI	StressLess Cardiovascular Dynamics Index
SD	Standard Deviation
bpm	Beats Per Minute
