# Supplementary material for: Short-Term Cardiovascular Changes During the StressLess University Dog-Assisted Wellbeing Program: Evaluation of Integrated Cardiovascular Indices
Source: Healthcare (Basel). 2026 Aug 11;14(16):2494. doi: 10.3390/healthcare14162494 (PMC13512088; doi:10.3390/healthcare14162494)
Supplement: Supplementary file 1 [file healthcare-14-02494-s001.zip › healthcare-4394085-supplementary.pdf]

# Supplementary Materials

## Short-Term Cardiovascular Changes during the StressLess University Dog-Assisted Wellbeing Program: Evaluation of Integrated Cardiovascular Indices

José Gijón, Meriem Khaled, María J. Lirola, and Miguel C. Botella

### Contents

Supplementary Figure S1. Representative StressLess sessions conducted in the university-library setting.

Supplementary Figure S2. Mean-difference plots for systolic blood pressure, diastolic blood pressure, and heart rate.

*All figures correspond to the study dataset and intervention setting described in the main manuscript.*

# Supplementary Figure S1

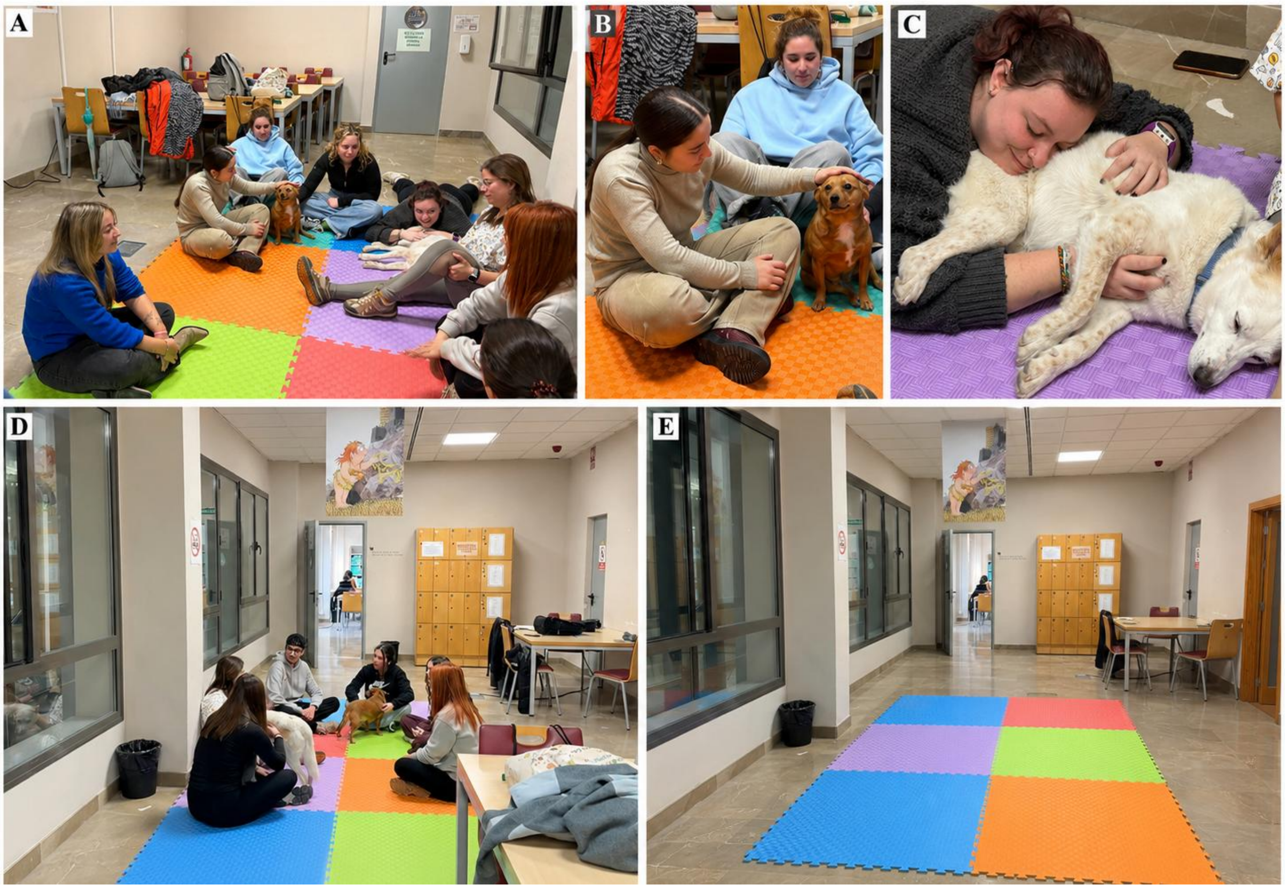


**Figure S1.** Representative StressLess sessions conducted in the reserved university-library room. Panels show (A) a small-group session; (B) supervised interaction with a trained dog; (C) calm physical contact with another trained dog; (D) a second supervised group session; and (E) the prepared room before a session. Written informed consent for publication of identifiable images was obtained from all individuals shown.

## Supplementary Figure S2

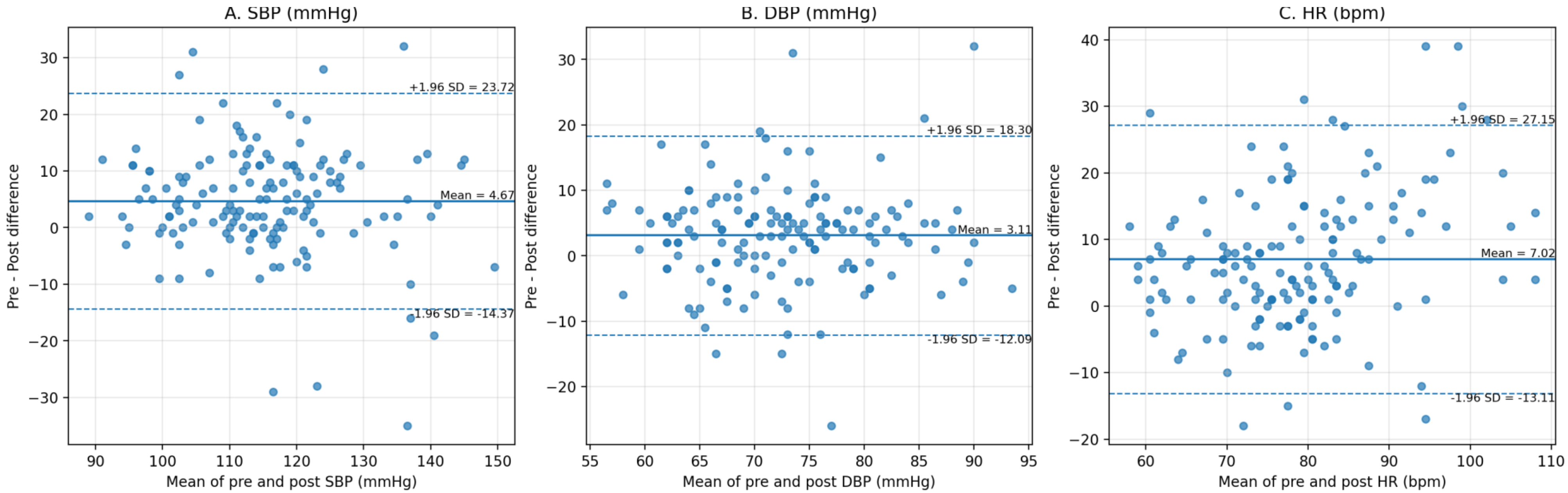


**Figure S2.** Mean-difference plots of cardiovascular measurements at participants' first attendance (N = 147). The pre–post difference is plotted against the mean of the pre- and post-session measurements for (A) systolic blood pressure, (B) diastolic blood pressure, and (C) heart rate. The solid horizontal line represents the mean difference, and the dashed lines represent the 95% limits of agreement. Positive differences indicate lower values after the session. SBP = systolic blood pressure; DBP = diastolic blood pressure; HR = heart rate; bpm = beats per minute.
